# Risk factors of HIV and variation in access to clean needles among people who inject drugs in Pakistan

**DOI:** 10.1080/20477724.2023.2191234

**Published:** 2023-03-22

**Authors:** Fernando Capelastegui, Adam Trickey, Laura H. Thompson, Tahira Reza, Faran Emmanuel, Francois Cholette, James F. Blanchard, Chris Archibald, Peter Vickerman, Aaron G. Lim

**Affiliations:** a Population Health Sciences, Bristol Medical School, University of Bristol, Bristol, UK; b Centre for Global Public Health, University of Manitoba, Winnipeg, Canada; c Canada-Pakistan HIV/AIDS Surveillance Project, Centre for Global Public Health, Islamabad, Pakistan; d National HIV and Retrovirology Laboratories, JC Wilt Infectious Diseases Research Centre, Public Health Agency of Canada, Winnipeg, Canada; e Department of Medical Microbiology and Infectious Diseases, University of Manitoba, Winnipeg, Canada; f Centre for Communicable Diseases and Infection Control, Public Health Agency of Canada, Ottawa, Canada

**Keywords:** HIV epidemic, PWID, IBBS, NSP, risk-group

## Abstract

We identified key risk factors for HIV among people who inject drugs (PWID) in Pakistan and explored access to free clean needles. Multivariable logistic regression was used to investigate associations between HIV prevalence and demographic, behavioral, and socio-economic characteristics of PWID. Data came from the Government of Pakistan’s Integrated Biological and Behavioral Surveillance (IBBS) Round 5 (2016–17; 14 cities). A secondary analysis investigated associations with reported access to clean needles. Unweighted HIV prevalence among 4,062 PWID (99% male) was 21.0%. Longer injecting duration (Odds ratio [OR] 1.06 [95% confidence interval: 1.02–1.10]; per year), higher injecting frequency (OR 1.67 [1.30–2.13]; per unit increase), and injecting heroin (OR 1.90 [1.11–3.25]) were positively associated with HIV prevalence. There was no association between using a used syringe at last injection and HIV. Having>10 years of education had lower odds of HIV than being illiterate (OR 0.58 [0.35–0.95]). Having a regular sexual partner (OR 0.74 [0.57–0.97]) or paying for sex with the opposite sex (OR = 0.62 [0.45–0.85]) had lower odds of HIV than not. Conversely, PWID paying a man/hijra for sex had higher odds of HIV (OR 1.20 [1.00–1.43]). Receipt of clean needles varied by city of residence (0–97% coverage), whilst PWID with knowledge of HIV service delivery programs had higher odds of receiving clean needles (OR 4.58 [3.50–5.99]). Injecting behaviors were associated with HIV prevalence among PWID, though risks related to paying for sex remain complicated. Geographical variation in access to clean needles suggests potential benefits of more widely spread public health services.

## Summary

Key Messages

What is already known on this topic

The HIV epidemic in Pakistan is concentrated among key populations including people who inject drugs.

What this study adds

Injecting practices, sexual behaviors, and socio-economic factors are associated with HIV prevalence among people who inject drugs. Access to harm reduction services is varied in Pakistan.

How this study might affect research, practice, or policy

Access to clean free needles, as well as service delivery programs, with a broad geographical reach remain important to curb the HIV epidemic among people who inject drugs in Pakistan.

## Background

In Pakistan, HIV is concentrated in key populations, including people who inject drugs (PWID), men who have sex with men (MSM), sex workers, and transgender communities. HIV prevalence is markedly higher in these key populations than that in the general population (0.1%) [1]. Repeated integrated biological and behavioral surveillance (IBBS) conducted by the Government of Pakistan across five rounds between 2005 and 2017 have suggested increasing HIV prevalence in all key populations [2–6] and overlapping risk factors are likely to play a role in this increase, which has also been suggested by previous modeling studies [7,8]. PWID is a key community of concern as historic epidemics have flared from within this population through sharing of injecting paraphernalia [9]. In the early 2000s, HIV prevalence among PWID in Pakistan was estimated to be approximately 20% [10], with recent surveillance data from 2016/17 estimating HIV prevalence has risen to 38% [11].

Prompt and effective interventions for PWID have been shown to curb HIV epidemics and include a variety of approaches [12,13]. Harm reduction services may include education, HIV testing, treatment clinics, needle exchanges [14], and opioid substitution therapy [15]. Alongside IBBS data collection, the Government of Pakistan has been running HIV prevention programs (called Service Delivery Programs, SDPs) since 2004, to help inform guidance and management of HIV in Pakistan. Despite this, according to the UNAIDS in recent years provision and access to services has faltered in Pakistan due to political events and a lack of national commitment to tackling the HIV epidemic [1,16]. This is supported by a previous study comparing IBBS data between rounds 2 (2006) and 4 (2011), where the reported use of a used needle during the last time injecting rose from 29.1% to 37.9% [17].

The aim of this study is to identify the main demographic, behavioral, and socio-economic status factors associated with HIV infection among PWID in Pakistan. Previous studies in Pakistan have carried out similar work, namely Archibald et al. [17], who identified key associations of HIV in PWID from Punjab and Sindh provinces using IBBS data from 2006 (round 2) [5] and 2011 (round 4) [4]. We use data from the latest round in 2016–17 (round 5) [3] to investigate associations and highlight any differences from previous rounds. A secondary aim is to provide exploratory insights into services uptake for PWID by investigating the factors associated with receipt of free, clean needles in the last year. This contributes to the understanding of the current HIV epidemic situation in Pakistan among PWID, with the aim of guiding harm reduction services to best control the epidemic.

## Methods

### Data sources

Data for this study were obtained from IBBS round 5 conducted by the National AIDS Control Program [2] among PWID across 14 cities of Pakistan between 2016 and 2017 (*n* = 4,062). Any person over the age of 13 years who injected drugs regularly for non-therapeutic reasons in the past six months was eligible for the study. Participants were recruited through identification of the top 10 hot spots of PWID within each city. HIV status was determined by a HIV rapid test. Alongside HIV testing, a behavioral questionnaire was undertaken, which comprised seven sections covering questions on: socio-demographic characteristics; drug and injection-related behaviors; sexual behaviors; knowledge and avoidance of AIDS/HIV; knowledge about sexually transmitted infections (STIs); harassment, discrimination, violence, and other risk behaviors; and services uptake. Further details on data collection can be found in the supplementary materials.

### Statistical analyses

Age-adjusted and multivariable logistic regression models were used to assess associations of demographic, behavioral, and socio-economic variables with HIV infection. Several variables identified as important HIV risk factors in analyses of earlier IBBS datasets among PWID in Pakistan [17] were selected a priori for inclusion in the final multivariable model. These include age, duration of injecting habit, number of injections on the previous day, location of last injection, the type of person they last injected with (i.e. family member, friend, sexual partner etc.), whether the last injection was with a used syringe, paying to have sex (with the opposite sex), having ever exchanged sex for money or drugs, and paying a man or hijra for sex in the past 6 months (asked to male PWID). To investigate associations between socio-economic variables and HIV status, variables on income and education were also included a priori. Regression models for each other variable in the dataset were carried out to assess univariable associations with HIV infection, adjusting solely for age. Variables were then selected using a threshold of *p* < 0.05 for the subsequent multivariable logistic regression model. To account for the geographic variance in data collection, the standard errors of the regression models were clustered by city (*n* = 14). In a post-hoc subgroup analysis to investigate the association between use of a used syringe and age on HIV, we restricted the sample to PWID aged<30 years old.

As a secondary analysis, a multivariable logistic regression analysis was carried out to test for associations of being provided with free, clean needles in the past year with demographic, behavioral, and socio-economic variables. Key demographic factors including age, income, education level, geographic location, language, prior knowledge of services, and type of drug injected were included in the model.

All statistical and descriptive analyses were carried out using STATA software version 15 [18]. The supplementary materials contain further details on data selection and statistical methods.

## Results

### Baseline characteristics

Table 1 outlines demographic characteristics of PWID participants from IBBS round 5. In total, 4,062 participants were enrolled and consisted of mainly male participants (*n* = 4,039; 99.4%). The unweighted HIV prevalence was 21.0% among participants. Most participants were aged 20–39 years (80.0%), whilst 40.1% of respondents reported being illiterate, with only 34.7% of participants having ≥6 years of education. The most common drugs injected by participants were heroin (*n* = 3213, 79.0%) and the antihistamine avil (*n* = 3207, 79.1%). Poly-substance use was common; 77.2% of participants reported injecting at least 2 substances.

**Table 1.** Demographic factors overall, stratified by HIV status, and by access to free, clean needles.

Variable		N	N (%) HIV-positive	N (%) Reported no access to clean, free needles	N (%) Reported access to clean, free needles	N (%) missing data on access to clean, free needles
Total		4,062	851 (21.0)	2158 (53.1)	1744 (42.9)	160 (3.9)
HIV Status	Negative	3,211	0 (0.0)	1837 (57.2)	1237 (38.5)	137 (4.3)
	Positive	851	851 (100.0)	321 (37.7)	507 (59.6)	23 (2.7)
Age category	<19	125	21 (16.8)	92 (73.6)	31 (24.8)	2 (1.6)
	20–29	1,689	305 (18.1)	991 (58.7)	627 (37.1)	71 (4.2)
	30–39	1,559	343 (22.0)	827 (53.0)	663 (42.5)	69 (4.4)
	40–49	510	143 (28.0)	205 (40.2)	292 (57.3)	13 (2.5)
	50–59	120	30 (25.0)	37 (30.8)	78 (65)	5 (4.2)
	60–69	44	7 (15.9)	4 (9.1)	40 (90.9)	0 (0.0)
	≥70	13	2 (15.4)	1 (7.7)	12 (92.3)	0 (0.0)
	Unknown	2	0 (0.0)	1 (50.0)	1 (50.0)	0 (0.0)
Income quintile	1	1,021	207 (20.3)	675 (66.1)	326 (31.9)	20 (2.0)
	2	548	112 (20.4)	253 (46.2)	275 (50.2)	20 (3.6)
	3	746	184 (24.7)	336 (45.0)	386 (51.7)	24 (3.2)
	4	807	185 (22.9)	396 (49.1)	395 (48.9)	16 (2.0)
	5	716	140 (19.6)	326 (45.5)	332 (46.4)	58 (8.1)
	Unknown	224	23 (10.3)	172 (76.8)	30 (13.4)	22 (9.8)
Education Category	Illiterate	1,627	392 (24.1)	854 (52.5)	709 (43.6)	64 (3.9.0)
	Up to 5 Yrs	994	200 (20.2)	574 (57.7)	388 (39.0)	32 (3.2)
	6 to 10 Yrs	1,233	231 (18.7)	604 (49.0)	575 (46.6)	54 (4.4)
	>10 Yrs	176	20 (11.4)	112 (63.6)	54 (30.7)	10 (5.7)
	Unknown	32	8 (25.0)	14 (43.8)	18 (56.3)	0 (0.0)
City	Rawalpindi	302	65 (21.5)	58 (19.2)	244 (80.8)	0 (0.0)
	Bhahwalpur	292	73 (25.0)	111(38.0)	173 (59.2)	8 (2.7)
	Kasur	302	153 (50.7)	6 (2.0)	293 (97.0)	3 (1.0)
	Jehlum	302	54 (17.9)	29 (9.6)	270 (89.4)	3 (1.0)
	Karachi	302	147 (46.7)	121 (40.1)	181 (59.9)	0 (0.0)
	Hyderabad	302	40 (13.3)	274 (90.7)	24 (7.9)	4 (1.3)
	Sukkur	302	50 (16.6)	217 (71.9)	61 (20.2)	24 (7.9)
	Larkana	302	49 (16.2)	295 (97.7)	6 (2.0)	1 (0.3)
	Nawabshah	302	40 (13.3)	209 (69.2)	90 (29.8)	3 (1.0)
	Mirpurkhas	302	70 (23.2)	32 (10.6)	268 (88.7)	2 (0.7)
	Peshawar	302	30 (9.9)	280 (92.7)	0 (0.0)	22 (7.3)
	Bannu	146	5 (3.4)	113 (77.4)	5 (3.4)	28 (19.2)
	Quetta	302	25 (8.3)	221 (73.2)	75 (24.8)	6 (2.0)
	Turbat	302	50 (16.6)	182 (60.3)	54 (17.9)	66 (21.9)
Mother tongue	Urdu	651	122 (18.7)	363 (55.8)	274 (42.1)	14 (2.2)
	Punjabi	1,156	339 (29.3)	287 (24.8)	855 (74.0)	14 (1.2)
	Sindhi	668	129 (19.3)	508 (76.0)	136 (20.4)	24 (3.6)
	Pashto	617	77 (12.5)	459 (74.4)	123 (19.9)	35 (5.7)
	Other	970	184 (19.0)	541 (55.8)	356 (36.7)	73 (7.5)
Injecting Tamgesic	No	3,926	831 (21.2)	2154 (54.9)	1707 (43.5)	65 (1.7)
	Yes	116	16 (13.8)	74 (63.8)	30 (25.9)	12 (10.3)
	Unknown	20	4 (20.0)	13 (65.0)	7 (35.0)	0 (0.0)
Injecting Avil	No	835	152 (18.2)	434 (52.0)	362 (43.4)	39 (4.7)
	Yes	3,207	695 (21.7)	1711 (53.4)	1375 (42.9)	121 (3.8)
	Unknown	20	4 (20.0)	13 (43.3)	17 (56.7)	0 (0.0)
Injecting Diazepam	No	3,406	725 (21.3)	1695 (49.8)	1563 (45.9)	148 (4.3)
	Yes	636	122 (19.2)	450 (70.8)	174 (27.4)	12 (1.9)
	Unknown	20	4 (20.0)	13 (65,0)	7 (35.0)	0 (0.0)
Injecting Buperon	No	3,814	797 (20.9)	2003 (52.5)	1656 (43.4)	155 (4.1)
	Yes	228	50 (21.9)	142 (62.3)	81 (35.5)	5 (2.2)
	Unknown	20	4 (20.0)	13 (65.0)	7 (35.0)	0 (0.0)
Injecting Heroin	No	829	91 (11.0)	518 (62.5)	258 (31.1)	53 (6.4)
	Yes	3,213	756 (23.5)	1627 (50.6)	1479 (46.0)	107 (3.3)
	Unknown	20	4 (20.0)	13 (65.0)	7 (35.0)	0 (0.0)
Injecting Sosegon	No	3,721	774 (20.8)	1962 (52.7)	1618 (43.5)	141 (3.8)
	Yes	321	73 (22.7)	183 (57.0)	119 (37.1)	19 (5.9)
	Unknown	20	4 (20.0)	13 (65.0)	7 (35.0)	0 (0.0)
Injecting Pentazegon	No	3,965	831 (21.0)	2102 (53)	1708 (43.1)	155 (3.9)
	Yes	77	16 (20.8)	43 (55.8)	29 (37.7)	5 (6.5)
	Unknown	20	4 (20.0)	13 (65.0)	7 (35.0)	0 (0.0)
Injecting Pentonil	No	3,982	835 (21.0)	2127 (53.4)	1698 (42.6)	157 (3.9)
	Yes	60	12 (20.0)	18 (30.0)	39 (65.0)	3 (5.0)
	Unknown	20	4 (20.0)	13 (65.0)	7 (35.0)	0 (0.0)
Injecting Pheneregan	No	3,866	781 (20.2)	2122 (54.9)	1586 (41.0)	158 (4.1)
	Yes	176	66 (37.5)	23 (13.1)	151 (85.8)	2 (1.1)
	Unknown	20	4 (20.0)	13 (65.0)	7 (35.0)	0 (0.0)
Duration injecting	1 year or less	692	65 (9.4)	421 (60.8)	251 (36.3)	20 (2.9)
	2 to 4 years	1,707	285 (16.7)	996 (58.3)	640 (37.5)	71 (4.2)
	5 to 9 year	1,017	322 (31.7)	461 (45.3)	526 (51.7)	30 (2.9)
	10 to 19 year	471	125 (26.6)	206 (43.7)	242 (51.4)	23 (4.9)
	20 year or more	129	46 (35.7)	47 (36.4)	77 (59.7)	5 (3.9)
	Unknown	46	8 (17.4)	27 (58.7)	8 (17.4)	11 (23.9)
Last time injected by professional injector	Yes	1,497	386 (25.8)	746 (49.8)	723 (48.3)	28 (1.9)
	No	2,456	455 (18.5)	1344 (54.7)	1007 (41.0)	103 (4.2)
	Unknown	111	10 (9.0)	68 (61.3)	14 (12.6)	29 (26.1)
Do you know where to get tested for HIV	No	1,315	191 (14.5)	861 (65.5)	411 (31.3)	43 (3.3)
	Yes	1,660	523 (31.5)	553 (33.3)	1075 (64.8)	32 (1.9)
	Unknown	1,087	137 (12.6)	744 (68.4)	258 (23.7)	85 (7.8)
Have you heard of programs for PWID, provided by the government of Pakistan?	No	2,196	333 (15.2)	1563 (71.2)	494 (22.5)	139 (6.3)
	Yes	1,866	518 (27.8)	595 (31.9)	1250 (67.0)	21 (1.1)

### Factors associated with HIV infection

Table 2 displays univariable and multivariable associations between risk factors and HIV infection. In multivariable analyses, injecting heroin was associated with a higher odds of HIV infection than not injecting heroin (OR 1.90, 95% CI 1.11–3.25; *p* = 0.020). PWID that were aware of HIV prevention programs (i.e. SDPs) had greater odds of having HIV than those that were unaware (OR 1.88, 95% CI 1.50–2.37, *p* < 0.001). Increased frequency of injection was strongly associated with HIV infection (OR 1.67, 95% CI 1.30–2.13; *p* < 0.001, per unit increase per day), as was an increased duration of injecting, with the risk of HIV rising by a factor of 1.06 times with every year of injecting (95% CI 1.02–1.10; *p* = 0.007). Having used a used syringe at last injection showed a positive univariable association with HIV infection, but there was no strong evidence of an association in multivariable analyses (OR 1.49, 95% CI 0.87–2.55; *p* = 0.143). In a subgroup analysis restricted to PWID aged<30 years old, the corresponding OR was 1.60 (95% CI 0.94–2.67; *p* = 0.082), indicating a stronger association between use of a used syringe and HIV, although the confidence intervals were wide.

**Table 2.** Age-adjusted (AA) and multivariable (MV) odds ratios (OR) with HIV infection.

		N (%) HIV positive	AA model OR (95% CI)	UV model p-value	MV model OR (95% CI)	MV model p-value
HIV status		851 (21.0)				
Age (per 1-year increase)			1.02 (1.01–1.03)	<0.001	0.99 (0.97–1.01)	0.234
Education level	Illiterate	392 (24.1)	1	Ref	1	Ref
	Up to 5 years	200 (20.1)	0.81 (0.66–0.98)	0.028	1.02 (0.82–1.27)	0.886
	6 to 10 years	231 (18.7)	0.74 (0.62–0.89)	0.001	0.88 (0.71–1.11)	0.860
	>10 Years	20 (11.4)	0.40 (0.25–0.65)	<0.001	0.58 (0.35–0.95)	0.030
Income quintile	1	207 (20.3)	1	Ref	1	Ref
	2	112 (20.4)	1.02 (0.79–1.31)	0.890	0.87 (0.59–1.28)	0.473
	3	184 (24.7)	1.29 (1.03–1.61)	0.027	1.07 (0.74–1.54)	0.732
	4	185 (22.9)	1.17 (0.93–1.46)	0.174	1.42 (0.94–2.13)	0.095
	5	140 (19.6)	0.93 (0.73–1.19)	0.579	1.14 (0.69–1.86)	0.613
Where do you live	Family home	473 (18.7)	1	Ref	1	Ref
	Dera (Male meeting place)	113 (5.7)	0.85 (0.47–1.55)	0.598	1.03 (0.49–2.18)	0.937
	Shrine	111 (31.4)	1.86 (1.45–2.39)	<0.001	1.13 (0.50–2.52)	0.773
	Street/lane	202 (22.9)	1.28 (1.06–1.54)	0.010	1.14 (0.76–1.76)	0.514
	Hotel/Hostel	11 (21.2)	1.18 (0.62–2.31)	0.626	1.82 (0.82–4.04)	0.142
	Quarter/flat	41 (26.6)	1.59 (1.10–2.31)	0.014	2.16 (1.03–4.53)	0.042
How long have you been injecting (per 1-year increase)			1.08 (1.06–1.10)	<0.001	1.06 (1.02–1.10)	0.007
Do you inject heroin?	No	91 (11.0)	1	Ref	1	Ref
	Yes	756 (23.5)	2.43 (1.93–3.07)	<0.001	1.90 (1.11–3.25)	0.020
How many times did you inject yesterday (per increase of 1)			1.80 (1.68–1.92)	<0.001	1.67 (1.30–2.13)	<0.001
Where did you last inject?	Own House/friend’s house	97 (16.0)	1	Ref	1	Ref
	Park/open space/Street	573 (21.4)	1.43 (1.13–1.81)	0.003	1.13 (0.72–1.78)	0.605
	Shrine	132 (29.0)	2.04 (1.51–2.75)	<0.001	1.00 (0.55–1.80)	0.996
	Hotel/Shop	31 (13.7)	0.84 (0.54–1.29)	0.422	0.75 (0.36–1.53)	0.425
	Work/graveyard/fruit market	11 (35.5)	2.95 (1.37–6.37)	0.006	2.40 (1.00–5.76)	0.050
With who did you inject last?	Alone	184 (16.8)	1	Ref	1	Ref
	Family members	7 (17.5)	1.05 (0.46–2.41)	0.909	1.50 (0.62–3.65)	0.371
	Friends	627 (23.1)	1.51 (1.26–1.81)	<0.001	1.14 (0.78–1.67)	0.509
	Drug seller	19 (16.8)	0.97 (0.58–1.63)	0.911	0.97 (0.46–2.03)	0.928
	Sexual partner	2 (5.0)	0.28 (0.07–1.18)	0.082	0.35 (0.06–2.13)	0.253
	Stranger	9 (28.1)	1.88 (0.85–4.13)	0.099	1.07 (0.43–2.64)	0.886
Last time you injected did you use a used syringe?	No	558 (19.0)	1	Ref	1	Ref
	Yes	259 (28.4)	1.65 (1.39–1.96)	<0.001	1.49 (0.87–2.55)	0.143
In the past 6 months have you had sex with a regular partner?	No	450 (28.5)	1	Ref	1	Ref
	Yes	286 (17.5)	0.54 (0.93–1.56)	<0.001	0.74 (0.57–0.97)	0.030
In the past 6 months have you paid to have sex with any males/females?	No	534 (25.8)	1	Ref	1	Ref
	Yes	204 (17.7)	0.64 (0.53–0.77)	<0.001	0.62 (0.45–0.85)	0.003
Have you ever exchanged sex for money or drugs?	No	483 (22.9)	1	Ref	1	Ref
	Yes	246 (23.0)	1.06 (0.88–1.26)	0.549	1.10 (0.77–1.55)	0.607
In the past 6 months have you ever paid a man or hijra to have sex with you?	No	518 (22.9)	1	Ref	1	Ref
	Yes	218 (22.7)	1.05 (0.87–1.26)	0.625	1.20 (1.00–1.43)	0.044
Have you taken alcohol in the course of a sexual act in the past 12 months?	No	726 (22.8)	1	Ref	1	Ref
	Yes	99 (14.3)	0.59 (0.47–0.74)	<0.001	0.68 (0.53–0.88)	0.003
Have you heard of programs for PWID, provided by the government of Pakistan?	No	333 (15.2)	1	Ref	1	Ref
	Yes	518 (27.8)	2.10 (1.80–2.45)	<0.001	1.88 (1.50–2.37)	<0.001

CI: Confidence interval. PWID: People who inject drugs.

Certain sexual behaviors were also strongly associated with reduced HIV infection status. Having sex with a regular partner (versus not) was associated with reduced risk of HIV (OR 0.74, 95% CI 0.57–0.97; *p* = 0.030), as was consuming alcohol during sexual intercourse (OR 0.68, 95% CI 0.53–0.88; *p* = 0.003). For male PWID, paying to have sex with other males/Hijras was associated with a higher risk of HIV (OR 1.20, 95% CI 1.00–1.43; *p* = 0.044) than those who did not. Meanwhile, those who reported paying for sex with the opposite sex, i.e. male PWID paying a female for sex, had lower odds of HIV infection than those that did not report this (OR 0.62, 95% CI 0.45–0.85; *p* = 0.003).

**Figure 1.**
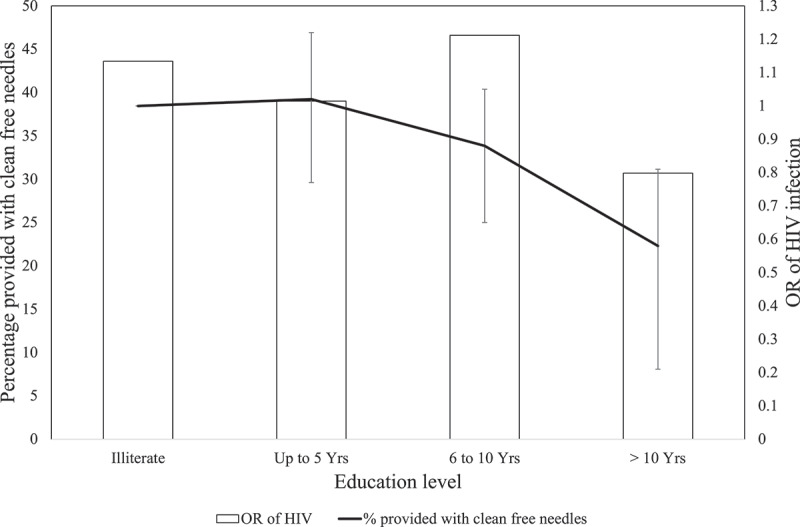
Percentage of PWID who report access to free, clean needles in the past year, and mean odds ratios (OR), with 95% confidence interval, of HIV infection for education levels versus being illiterate.

Regarding socio-economic variables, higher education was negatively associated with HIV infection (Figure 1). Having over 10 years of education was strongly associated with a reduced risk of HIV infection (OR 0.58, 95% CI 0.35–0.95; *p* = 0.030) versus being illiterate. Those living in a flat were at higher risk of HIV compared to living in a family home (OR 2.16, 95% CI 1.03–4.53; *p* = 0.042).

### Factors associated with access to free needles

**Figure 2.**
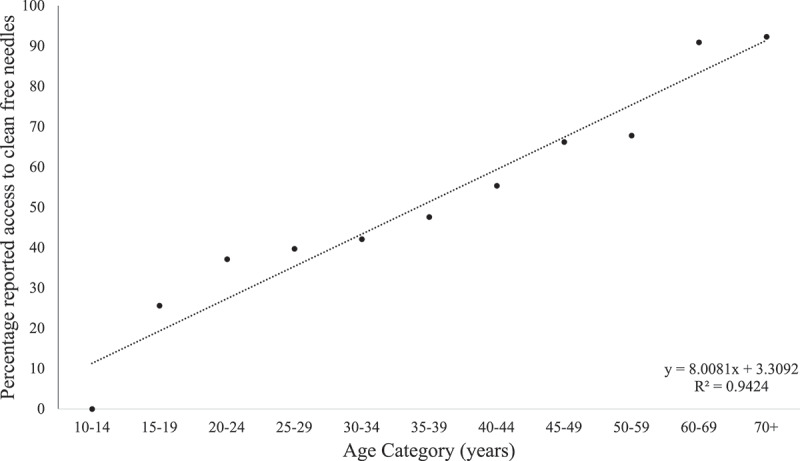
Percentage of PWID reporting having been provided with free, clean needles in the last year by age category.

Table 3 outlines the results of the regression model investigating the variables associated with access to free, clean needles over the past year. Being HIV positive was not associated with access to needles (OR 1.05, 95% CI 0.78–1.41; *p* = 0.765), whereas the odds of receiving free clean needles rose by a factor of 1.02 (95% CI 1.01–1.04; *p* = 0.006) per year increase in age (Figure 2). Those in the 2^nd^ and 3^rd^ income quintiles were at least twice as likely to report access to clean needles (OR 2.41, 95% CI 1.60–3.61; *p* < 0.001 and OR 1.98, 95% CI 1.39–2.84; *p* < 0.001, respectively) as those in the 1^st^ quintile.

**Table 3.** Univariable and multivariable odds ratios for associations with the receipt of free, clean needles in the prior year.

Variable		N (%) receiving free, clean needles	UV model OR (95% CI)	UV model p-value	MV model OR (95% CI)	MV model p-value
HIV	Negative	1237 (40.2%)	1	Ref	1	Ref
	Positive	507 (61.2%)	2.35 (2.00–1.75)	<0.001	1.05 (0.78–1.41)	0.765
Age	Per year increase	NA	1.05 (1.04–1.06)	<0.001	1.02 (1.01–1.04)	0.006
Income quintile	1	326 (32.6%)	1	Ref	1	Ref
	2	275 (52.1%)	2.25 (1.81–2.79)	<0.001	2.41 (1.60–3.61)	<0.001
	3	386 (53.5%)	2.38 (1.95–2.90)	<0.001	1.98 (1.39–2.84)	<0.001
	4	395 (49.9%)	2.07 (1.70–2.50)	<0.001	1.30 (0.89–1.88)	0.171
	5	332 (50.5%)	2.11 (1.72–2.58)	<0.001	0.84 (0.56–1.25)	0.395
Education Category	Illiterate	709 (45.4%)	1	Ref	1	Ref
	Up to 5 Yrs	388 (40.3%)	0.81 (0.69–0.96)	0.013	0.97 (0.71–1.33)	0.853
	6 to 10 Yrs	575 (48.8%)	1.15 (0.99–1.33)	0.077	0.84 (0.63–1.12)	0.225
	>10 Yrs	54 (32.5%)	0.58 (0.41–0.82)	0.002	0.64 (0.37–1.09)	0.102
City	Rawalpindi	244 (80.8%)	1	Ref	1	Ref
	Bhahwalpur	173 (60.9%)	0.37 (0.26–0.54)	<0.001	0.40 (0.21–0.76)	0.005
	Kasur	293 (98.0%)	11.61 (4.92–27.36)	<0.001	5.61 (1.88–16.74)	0.002
	Jehlum	270 (90.3%)	2.21 (1.37–3.57)	0.001	2.58 (1.21–5.49)	0.014
	Karachi	181 (59.9%)	0.36 (0.25–0.51)	<0.001	0.32 (0.18–0.58)	<0.001
	Hyderabad	24 (8.1%)	0.02 (0.01–0.03)	<0.001	0.01 (0.00–0.02)	<0.001
	Sukkur	61 (21.9%)	0.07 (0.04–0.10)	<0.001	0.04 (0.02–0.10)	<0.001
	Larkana	6 (2.0%)	0.00 (0.00–0.01)	<0.001	0.00 (0.00–0.01)	<0.001
	Nawabshah	90 (30.1%)	0.10 (0.07–0.15)	<0.001	0.19 (0.10–0.34)	<0.001
	Mirpurkhas	268 (89.3%)	1.99 (1.26–3.17)	0.004	2.74 (1.38–5.46)	0.004
	Peshawar	0 (0.0%)	NA	NA	NA	NA
	Bannu	5 (3.9%)	0.01 (0.00–0.02)	<0.001	0.01 (0.00–0.14)	<0.001
	Quetta	75 (25.3%)	0.08 (0.05–0.12)	<0.001	0.07 (0.04–0.14)	<0.001
	Turbat	54 (22.9%)	0.07 (0.05–0.11)	<0.001	0.10 (0.05–0.21)	<0.001
Mother tongue	Urdu	274 (43.0%)	1	Ref	1	Ref
	Punjabi	855 (74.9%)	3.95 (3.21–4.85)	<0.001	1.28 (0.85–1.93)	0.232
	Sindhi	136 (21.1%)	0.35 (0.28–0.45)	<0.001	0.98 (0.60–1.58)	0.921
	Pashto	123 (21.1%)	0.36 (0.28–0.46)	<0.001	1.77 (1.05–2.99)	0.033
	Balochi	134 (33.3%)	0.66 (0.51–0.86)	0.002	1.13 (0.69–1.86)	0.627
	Bralvi	20 (20.4%)	0.34 (0.20–0.57)	<0.001	1.77 (0.85–3.67)	0.126
	Siraiki	155 (54.4%)	1.58 (1.19–2.09)	0.001	1.11 (0.67–1.83)	0.697
	Hindko	37 (37.4%)	0.79 (0.51–1.22)	0.291	1.34 (0.48–3.75)	0.572
	Other	10 (83.3%)	6.62 (1.44–30.48)	0.015	3.22 (0.5–20.73)	0.218
Injecting Tamgesic	No	1707 (45.2%)	1	Ref	1	Ref
	Yes	30 (28.9%)	0.49 (0.32–0.76)	0.001	0.96 (0.36–2.58)	0.936
Injecting Avil	No	362 (45.5%)	1	Ref	1	Ref
	Yes	1375 (44.6%)	0.96 (0.82–1.13)	0.641	0.88 (0.64–1.20)	0.408
Injecting Diazepam	No	1563 (47.7%)	1	Ref	1	Ref
	Yes	174 (28.9%)	0.45 (0.37–0.54)	<0.001	1.12 (0.75–1.66)	0.589
Injecting Buperon	No	1656 (45.3%)	1	Ref	1	Ref
	Yes	81 (36.3%)	0.69 (0.52–0.91)	0.010	0.84 (0.50–1.41)	0.509
Injecting Heroin	No	258 (33.3%)	1	Ref	1	Ref
	Yes	1479 (47.6%)	1.83 (1.55–2.15)	<0.001	0.86 (0.58–1.25)	0.425
Injecting Sosegon	No	1618 (45.2%)	1	Ref	1	Ref
	Yes	119 (39.4%)	0.79 (0.62–1.00)	0.052	0.63 (0.34–1.17)	0.141
Injecting Pentazegon	No	1708 (44.8%)	1	Ref	1	Ref
	Yes	29 (40.3%)	0.83 (0.52–1.33)	0.442	0.47 (0.17–1.30)	0.145
Injecting Pentonil	No	1698 (44.4%)	1	Ref	1	Ref
	Yes	39 (68.4%)	2.71 (1.55–4.76)	<0.001	3.52 (1.34–9.20)	0.01
Injecting Pheneregan	No	1586 (42.8%)	1	Ref	1	Ref
	Yes	151 (86.8%)	8.78 (5.64–13.69)	<0.001	1.53 (0.43–5.46)	0.514
Do you know where to get tested for HIV?	No	411 (32.3%)	1	Ref	1	Ref
	Yes	1075 (66.0%)	4.07 (3.48–4.76)	<0.001	1.64 (1.26–2.14)	<0.001
Have you heard of programs for PWID, provided by the government of Pakistan?	No	494 (24.0%)	1	Ref	1	Ref
	Yes	1250 (68.8%)	6.65 (5.78–7.65)	<0.001	4.58 (3.50–5.99)	<0.001

UV: Univariable. MV: Multivariable. OR: Odds ratio. CI: Confidence interval. PWID: People who inject drugs.

Geographically, there was a high variation in the percentage of PWID reporting being provided with free clean needles across cities (0.0–97.0%). Living in Kasur (OR 5.61, 95% CI 1.88–16.74; *p* = 0.002), Mirpurkhas (OR 2.74, 95% CI 1.38–5.46; *p* = 0.004) and Jhelum (OR 2.58, 95% CI 1.21–5.49; *p* = 0.014) was associated with higher access to free syringes/needles compared to living in Rawalpindi. Living in Karachi, the capital, was associated with lower reported access to needles (OR 0.32, 95% CI 0.18–0.58; *p* < 0.001) compared to Rawalpindi, as was the case for several other sampled cities, including Peshawar where 0% reported access to free needles.

Of the drugs, only injecting Pentonil (versus not) was associated with increased access to needles (OR 3.52, 95% CI 1.34–9.20; *p* = 0.01). Knowledge of where to get an HIV test (versus not knowing) was associated with access to needles (OR 1.64, 95% CI 1.26–2.14; *p* < 0.001) as was being aware (versus not) of SDPs (OR 4.58, 95% CI 3.50–5.99; *p* < 0.001).

## Discussion

Various risk factors and behaviors are associated with HIV among PWID in Pakistan. The prevalence of HIV among PWID (21.0%) from this study is in line with other research [1,2,17,19], which confirms the gravity of the HIV epidemic among PWID. Increased duration and frequency of injecting behavior were both found to be strongly associated with increased HIV risk, with a 1.06 increased odds of HIV infection for each additional year of injecting and 1.67 increased odds of HIV for each additional injection per day. These associations have been reported in previous analysis of IBBS datasets among PWID in Pakistan [17]. High risk injecting practices such as sharing and the use of dirty syringes put PWID at higher risk of HIV infection [20]. Our findings, however, did not show evidence that the use of a used syringe to inject was associated with having HIV, although there was stronger evidence among younger PWID. This lack of a strong association is surprising; however, similar work by Archibald et al. [17] returned the same result, suggesting that self-reported use of a used syringe is likely underreported and biased in PWID [21]. Findings from this study showed injecting heroin to be strongly associated with HIV, with the odds of HIV infection among those who injected heroin was 90% higher than those who did not. Injecting practices associated with heroin include sharing the same needle and ‘jerking’, whereby blood and heroin is mixed and deliberately shared between users [22–24]. In other studies, injecting diazepam or avil has been associated with HIV among PWID [17], particularly as they are often mixed with heroin in the same injection [25], although our study did not show that.

We also found associations between sex work and HIV. The odds of HIV infection in male PWID who reported paying other men and/or Hijras was 1.2 times higher compared to those who did not, supporting the presumption of high HIV transmission risk resulting from overlap between PWID and sex workers [26]. PWID are frequently involved with sex work as a means of income and to access drugs, and those who have sex with Hijras are at particularly high risk of HIV infection as Hijras are the group with the 2^nd^ highest HIV-prevalence in Pakistan [24,27]. However, an unexpected result is that among PWID who pay to have sex with the opposite sex, the odds of HIV infection is reduced by 38%. This goes against the convention that sex workers are a high-risk HIV population [28], although similar findings were also reported in previous analyses of Pakistan IBBS datasets [17]. This prompts the idea that individuals partaking in sex between men or Hijras are less aware of the risk of STI’s or HIV and wear protection less often, compared to those males partaking in sex with female sex workers. Additionally, we found that consumption of alcohol during sexual intercourse was negatively associated with HIV infection. Although the association of alcohol and prevalent HIV infection is yet to be fully understood [29], intoxication is usually associated with increased HIV infection due to fewer inhibitions and riskier behaviors [29,30]. Being a predominantly Muslim country, alcohol is prohibited, however this is a generalization as in some districts such as Isa Nagri (Karachi) there are large Christian and other religious minority populations, meaning questionnaire data relating to drinking habits may be complex to analyze at face value. Sex work is illegal in Pakistan [31], however, these findings are useful as an established sex work industry exists. The government provides limited service provision and education to sex worker communities [31], and addressing sex worker wellbeing is important for preventing the transmission of HIV.

Lower socio-economic status is linked to riskier sexual behaviors and higher engagement in drug use [32]. Higher HIV prevalence among poorer individuals is potentially seen because individuals may exchange sex for drugs and money and are more likely to have issues of substance dependence and addiction [28,32]. For example, our results show a high level of education is associated with a 42% reduced odds of HIV infection versus those that are illiterate. This may be due to better knowledge of HIV and prevention methods but could also be a more nuanced indicator of wealth and quality of life. Arguably, these are indicators of an individual’s wider socio-economic status alongside education and may provide an interesting platform for further investigation, particularly in Pakistan where a large proportion (24.3% in 2015) of individuals live in poverty [28,33].

Being aware of SDP availability is strongly associated with a higher risk of HIV infection. This may be because higher-risk individuals are more aware of services available to them and seek them out. In particular, those in higher risk categories of longer duration and higher frequency PWID may be more aware and dependent on needle exchanges. Alternatively, HIV-positive PWID may enter the care system to get tested or receive treatment and become aware of services available to them, though there is no straightforward conclusion from this finding.

Access to harm reduction services in Pakistan is limited. Previous IBBS rounds have found a low number of PWID accessing services. For example, in this round 5 (2016–2017), 24.6% of PWID reported using HIV services, which is a limited increase from 16.5% in 2006 [34]. Lahore and Karachi, both major cities, have experienced a greater burden of HIV and may have established higher levels and quality of services [35], aided by their importance and superior economic situations. The findings corroborate with the notion that there is variation between cities and provinces, which is expected. Although city and province population sizes have not been accounted for, the data suggest service provision and uptake are far from equal.

Although the results do not strongly indicate that younger individuals report less access to free needles in the last year, younger participants do make up a large proportion of PWID. Education and engagement with harm reduction services at a younger age is beneficial to reducing high-risk injecting behavior and to break the stigma associated with seeking services [36]. Targeting services at younger PWID should be a priority in Pakistan to reduce drug use alongside the risk of HIV infection from injecting with used needles. Anecdotally, this is shown as those who are aware of SDPs and where to get tested for HIV; in 2016–2017, 64.8% of participants knew where to get tested for HIV, and 67.0% of participants that have heard of SDPs also reported access to free, clean needles in the last year, which demonstrates a degree of effectiveness of harm reduction services.

A strength of this study is the nationwide coverage of the dataset across 14 cities, encompassing 4,062 PWID. Only 0.6% of the participants were female, however, females only make up a small percentage of drug users in Pakistan, so this is likely representative of the overall PWID population [37,38]. Furthermore, female PWID may be less willing to participate if male researchers are conducting the study [39]. Future studies enrolling more female PWID are needed to investigate how sex affects risk factors associated with HIV; although there may be few female PWID in this setting. Additionally, the sample sizes from each city were not necessarily representative. For example, there were similar numbers of participants from the capital Karachi (*n* = 302) with a population of 16 million [40], as there were from Kasur (*n* = 299), a smaller city with a population of approximately 382,000 [40]. Moreover, data on incarceration history were not collected in this survey, which is a limitation as high-risk behaviors including injecting drug use are higher in those with a history of prison episodes [41,42]. It is important for this variable to be accounted for in future studies.

Lastly, the questionnaire also contained data on discrimination and poor treatment of PWID, sex workers, and those who are HIV positive, which were beyond the scope of this analysis. It is well reported that stigmatization of HIV leads to worse outcomes in people living with HIV [43], and that issues of discrimination in healthcare settings persist [44], so it would be prudent to investigate these aspects next as a priority. This dataset provides invaluable insights into this angle of harm reduction services and would be useful to establish whether there are issues in providing services to these individuals fairly. Localized analysis of access to free clean needles or treatment for HIV could help target future action plans with the aim of reducing HIV transmission within marginalized communities and into the general community. Furthermore, this will be relevant not only to the management of HIV but other infections affecting PWID such as viral hepatitis as well as other sexually transmitted infections [45,46].

## Conclusions

PWID are an important community both globally and in Pakistan when considering HIV. Addressing high HIV prevalence among PWID and other marginalized groups is key to preventing further spread of HIV into the general population.

A novel aspect of this study is the focus on socio-economic risk factors on HIV infection which have not been fully explored in the past. This insight into the importance of SDP uptake by PWID and knowledge of SDP via reported access to clean-free needles provides an interesting perspective for future investigation. A holistic approach to HIV prevention is necessary and includes both education and physical services such as access to clean needles. Access to these services appears to be fragmented and sparse in some populations and regions in Pakistan, suggesting more could be done to improve the services available.

## Supplementary Material

Supplemental Material

## Data Availability

Due to the sensitive nature of this research, data analyzed in this study are not publicly available.

## List of abbreviations

AIDS – Acquired immunodeficiency syndrome

HIV – Human immunodeficiency virus

IBBS – Integrated biological and behavior survey

MV – Multivariable

PWID – People who inject drugs

SDP – Service delivery programme

STI – Sexually transmitted infection

UV – Univariable
