# Supplementary material for: Risk factors of HIV and variation in access to clean needles among people who inject drugs in Pakistan
Source: Pathog Glob Health. 2023 Mar 22;117(8):696–707. doi: 10.1080/20477724.2023.2191234 (PMC10614703; doi:10.1080/20477724.2023.2191234)
Supplement: Supplemental Material [file YPGH_A_2191234_SM7936.docx]

Appendix

Supplement to:

**Risk factors of HIV and variation in access to clean needles among people who inject drugs in Pakistan.**

**Further Details on Materials and Methods**

**HIV measure and questionnaire data collection**

The IBBS study methodology consisted of two phases: Phase 1 (Programmatic Mapping) estimated the size of selected key populations, including people who inject drugs (PWID), through geographic and network mapping in 14 different cities across Pakistan, which involved the identification of hot spots; Phase 2 (IBBS) determined the socio-demographic profiles, HIV risk behaviours, and HIV prevalence of these key populations to inform the HIV prevention programme in Pakistan^1^. Participants were enrolled through multi-stage cluster sampling from hot spots identified during the mapping phase, with recruitment of randomly selected participants facilitated by a social mobilizer. PWID participants were brought to the central office and were interviewed there. Consent was obtained prior to recruitment by the interviewers. Separate consent was also taken prior to HIV testing. Socio-behavioural data was collected by trained interviewers^1^.

The main outcome of the study, HIV status, was determined by an HIV test undertaken at the time of data collection in the field. This was achieved by collecting up to 2 samples which were then used for rapid testing. The first test used was an Alere Determine HIV-1/2 Ag/Ab Combo^1^. If this test returned a positive test, a further test using a Uni-Gold HIV kit was conducted. If the first test was negative, no further tests were carried out. As a measure of quality, 2% of negative tests and 5% of positive tests were stored and further analysed to ensure data collection and diagnoses were accurate. Those who refused a test were not included in the final dataset. All participants were offered support, especially in the case of a positive test where they were directly registered with an HIV support clinic^1^

Further details on materials and the IBBS study methodology can be found in the National AIDS Programme Integrated Biological & Behavioural Surveillance Round 5 report 2016-2017^1^.

**Data Formatting**

Within the original data provided, some variables were reformatted to improve the distribution of data more effectively within categories. For instance, income was split into quintiles to have an objective and even split of participants. Formatting and condensing of categorical variables also occurred for some variables which had sparsely populated categories. In the final multivariable (MV) model, the variable ‘with who did you last inject’ was recategorized from the initial format in the data set. This included combining responses ‘people I know well’ into the ‘friends’ category. The variable ‘where did you last inject’ was recategorized into the main categories displayed in the final model in a similar manner and included sorting answers from the ‘other’ response into the category of ‘work, graveyard, fruit market’. Meanwhile, age and frequency of injection were included as continuous variables.

**Variable selection:**

All the variables from the questionnaire had their association with HIV tested using a univariable (UV) model. Age was included in all of these UV models as a continuous variable. Age was included as it is a confounder, and adjustment was needed to account for increased HIV risk over time. This ensured the variable of interest is associated in its own right with HIV infection and not a product of age or years spent injecting.

Due to the scale of the questionnaire, only those variables selected in the UV model were used in the MV model if they had p-value<0.05. In addition, a priori variables were selected based on previous findings in the literature and were similar to the a priori variables selected in the research by Archibald et al^2^. These included income, education, duration of injecting habit, number of injections yesterday, location of last injection, person they last injected with, whether the last injection was with a used syringe, paying to have sex (with the opposite sex), having ever exchanged sex for money or drugs, and paying a man or hijra for sex in the past 6 months (asked to male PWID). In this analysis, income was included in the model a priori in order to explore the effect of socio-economic factors. Especially when linked to the descriptive analysis of access to clean needles, income was deemed a necessary variable to include.

When deciding between similar variables, the most recently framed question/data point was used. For example, ‘last time you injected was the syringe used’ rather than ‘in the past 6 months how many times did you use a used syringe’. This ensured there was limited collinearity between similar variables in the model, and also prevented recall bias from the participants, e.g. which may lead to inaccurate or conflicting answers to similar questions. This approach was used mainly in relation to questions relating to drug taking behaviour. Other areas this selection method was used was in relation to sexual behaviour. For example, where a question asked about condom usage ‘in the last 6 months’, or ‘during the most recent’ sexual activity, the most recent variable was selected.

**Descriptive analysis of access to clean needles.**

Variables in relation to participants reporting having been given clean needles for free were selected by hand. Only key demographic factors from the questionnaire were selected as well as variables related to knowledge of services. For example, it was assumed that knowing where to get tested for HIV was linked with being provided with clean needles, therefore, this variable was included as a point of interest. Knowing about services for PWID was also assumed to be linked to prior access of services and, because this variable was strongly associated with HIV infection in the final MV model for the HIV risk factor analysis, it was included to further understand the context of HIV infection and access to free, clean needles. Type of drug injected was also included in the analysis to explore whether there was a link with being provided with clean needles, particularly as heroin was found to be strongly associated with HIV in the MV model.

Age categories were created to examine the spread of ages in those reporting access to clean needles. Due to the distribution of ages, age categories were spread between 5- or 10-year spans, depending on the frequency of participants in each category. For example, above 50 years of age, the categories span 10 years as opposed to 5 years to reflect fewer participants in these age brackets.

**Appendix bibliography**

1. National AIDS Control Program. Integrated Biological & Behavioral Surveillance in Pakistan 2016-17. Islamabad, Pakistan: National Institute of Health, 2017.

2. Archibald CP, Shaw SY, Emmanuel F, et al. Geographical and temporal variation of injection drug users in Pakistan. *Sexually Transmitted Infections* 2013; **89**(Suppl 2): ii18-ii28.
